# Open Seldinger-guided peripheral femoro-femoral cannulation technique for totally endoscopic cardiac surgery

**DOI:** 10.1186/s13019-021-01584-x

**Published:** 2021-07-22

**Authors:** Yi Chen, Liang-wan Chen, Xiao-fu Dai, Xue-shan Huang

**Affiliations:** 1 Department of Cardiovascular Surgery, Union Hospital, Fujian Medical University, Fuzhou, China; 2 Key Laboratory of Cardio-Thoracic Surgery (Fujian Medical University), Fujian Province University, Fuzhou, China

**Keywords:** Seldinger-guided technique, Femoro-femoral cannulation, Totally endoscopic

## Abstract

**Background:**

The cannulation technique used in totally endoscopic cardiac surgery has a significant impact on the overall prognosis of patients. However, there are no large cohort studies to discuss it. Here we report on our research of using open Seldinger-guided technique to establish femoro-femoral cardiopulmonary bypass during totally endoscopic cardiac surgery and evaluate its safety and efficacy.

**Methods:**

The institutional database from 2017 to 2020 was retrospectively reviewed to find cases in which totally endoscopic cardiac surgery was performed. We identified 214 consecutive patients who underwent totally endoscopic cardiac surgery with peripheral femoro-femoral cannulation. All patients underwent femoral artery cannulation. Of these, 201 were cannulated in the femoral vein and 13 were cannulated in the femoral vein combined with internal jugular cannulation. The technique involves surgically exposing the femoral vessel, setting up purse-string over the vessels and then inserting a guidewire into the femoral vessel without a vascular incision, followed by exchange of the guidewire with a cannula.

**Results:**

Surgery indications included mitral valve disease in 82.71% (177/214), atrial septal defect in 11.68% (25/214) and tricuspid regurgitation in the remaining 5.61% (12/214). Hospital survival was 98.60% (211/214). There were no cases of stroke and postoperative limb ischaemia. No femoral vessel injuries or wound infections was observed. No late pseudoaneurysms were evident.

**Conclusion:**

The open Seldinger-guided femoro-femoral cannulation technique is effective and safe. We highly recommend this technique, given its safety, simplicity and speed under direct vision. The limited manipulation of the vessels under direct vision minimizes the risk of local complications.

**Supplementary Information:**

The online version contains supplementary material available at 10.1186/s13019-021-01584-x.

## Introduction

Minimally invasive cardiac surgery (MICS) was successfully introduced since the mid-1990 s [1, 2]. Due to the advantages of more attractive cosmetic results, lower pain levels, less physical trauma and quicker recovery, MICS has gradually become more and more popular worldwide [3–6].

Totally endoscopic cardiac surgery is one of the MICS, characterized by the non-disruption of the thoracic structures and the extra-thoracic extracorporeal circulation [3, 7, 8]. Previous studies have confirmed the safety and effectiveness of this procedure [6, 9].

In totally endoscopic cardiac surgery, perfusion strategies have a significant impact on the overall prognosis of the patient. It needs to meet the following conditions: 1. The flow should meet the needs of adequate perfusion without increasing neurological complications or vascular injuries; 2. Can be easily and fast manipulate; 3. The exposure is satisfied and the intracardiac visual field should not be affected. Besides, it also needs to reduce the risk of limb ischemia [10].

The most common perfusion strategies used during totally endoscopic cardiac surgery was the femoro-femoral cardiopulmonary bypass (CPB) in our institution. Open Seldinger-guided peripheral femoral cannulation was routinely used to establish extra-thoracic extracorporeal circulation [11].

Currently, the mainstream cannulation technique to establish femoro-femoral CPB included: percutaneous puncture [12, 13], traditional vascular cannulation technique [14], and using side-arm graft sewn to the femoral artery for perfusion [15]. To reduce complications related to lower limb ischemia, the bidirectional perfusion cannula has also been developed [16]. However, few literatures have reported on the specific steps of peripheral vascular cannulation to establish femoro-femoral cardiopulmonary bypass.

Our institutional preference is surgical exposure of the femoral vessels and then performed Seldinger-guided femoral artery cannulation [17], with venous drainage from the vena cava by femoral vein cannulation or cannulated in the femoral vein combined with internal jugular cannulation. For this approach, it is a technique that combines operational speed and safe under direct vision with minimal risk. The advantages of this technique are as follows:visibility, simplicity, speed and safe.

Since 2017, the open Seldinger-guided femoro-femoral cannulation has been routinely used in our institution. The aim of our study is to assess the efficacy and safety of this technique.

## Materials and methods

### Patients and methods

We retrospectively analyzed our institutional dataset from 2017 to 2020 to find all patients who underwent totally endoscopic cardiac surgery. This retrospective analysis was based on data from the Institute of Cardiothoracic Surgery at Fujian Union Hospital. In this study, informed consent was not required as the analysis is conducted using anonymous clinical data from each patient.

Medical records were specifically reviewed for different types of cardiac surgery (atrial septal defect repair, tricuspid valve repair, or mitral valve surgery), site of cannulation (femoral, jugular vein), and reasons for combined cannulation, post-operative data. It also includes demographic data, including gender, age, and body mass index. Special attention is paid to the complications associated with cannulation, including intra-operative arterial and venous injury (vascular rupture or artery dissection), lower limb ischemia, wound infection, hematoma or seroma.

### Surgical techniques

The cannulation procedure has been developed which is designed to combine efficiency and speed with minimal risks. The steps in the procedure include: (I) surgical exposure the femoral vessels; (II) peripheral femoro-femoral cannulation by the open Seldinger technique; (III) Transesophageal echocardiography (TEE) guided access and properly positioning of the cannula.

Preoperative examination was carefully performed to rule out possible contraindications such as severe calcifications in descending aorta and iliofemoral artery or patients with inferior vena cava filters or deep venous thrombosis. Femoral artery cannulation cannot be used when there is atherosclerosis or other potential source of embolism in the thoraco-abdominal aorta. Other reasons for not having femoro-femoral cannulation include extreme obesity or peripheral vascular disease of the iliac-femoral vascular system.

**Fig. 1:**
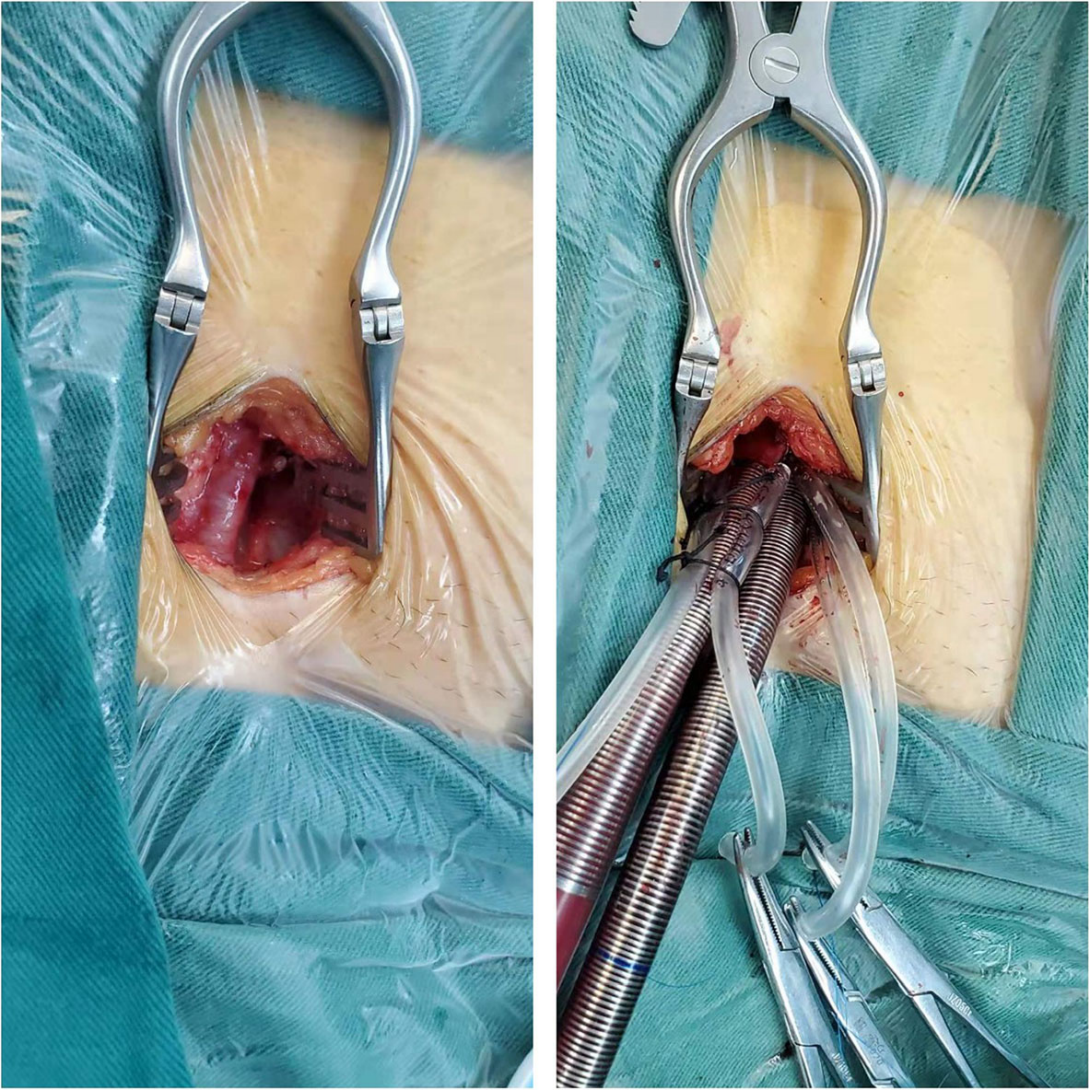
Surgical exposure and cannulation of femoral vessels

Open Seldinger-guided femoro-femoral cannulation was carried out as follows: First a vertical incision is made in the groin, through the inguinal crease. We usually prefer the right side, unless there is evidence of a lesion in the ipsilateral right femoral vessel, in which case we choose the left groin. The surgeons were divided into two groups, one for chest wall ports made and one for femoro-femoral cannulation. Dissecting the subcutaneous tissue to expose the femoral sheath, which was incised longitudinally. Isolated the femoral vessels from the surrounding tissue using a sharp dissection. Two independent purse-string sutures were made on the exposed anterior femoral vessels using 5–0 Prolene. Following administration of heparin, an 18G needle was then inserted into the purse-string, a 0.038-in. guidewire was guided through the needle into the thoracic descending aorta or inferior vena cava under TEE guidance. We use a femoral arterial cannula of 18Fr to 22Fr and a single two-stage venous cannula of 24Fr to 28Fr (this depends on the patient’s weight). This arterial and venous cannula with a tapered tip accommodates the guidewire has a smooth transition from the dilator to the cannula for easy insertion. After removal of the needle, the dilator and the cannula were advanced over the guidewire with the modified Seldinger-technique technique. When correctly positioned under TEE, the internal dilator is removed, the cannula is connected to the arterial circuit and venous circuit and the cannula secured to the snare by cinched the purse-string down. With femoral or combined venous cannulation is performed, femoro-femoral CPB is initiated. After the operation is ended, when the patient is weaned from extracorporeal circulation, the cannula was removed, usually by simply tied the purse-string and no clamping the vessel. We routinely made an additional purse-string around the first purse-string to reduce the risk of bleeding. Then check for an arterial pulse at the distal end of the purse-string (Fig. 1).

### Definition of clinical parameters

Mortality was defined as all-cause death less than 30 days after surgery or during hospitalization. Stroke was defined as an acute episode of focal dysfunction of the brain, retina, or spinal cord lasting longer than 24 h, or of any duration if imaging (CT or MRI) or autopsy show focal infarction or haemorrhage relevant to the symptoms [18, 19]. The definition of acute kidney injury is based on the Kidney Disease Improving Global Outcomes (KDIGO) criteria [20].

### Statistical analysis

SPSS (IBM, Version 24.0) was used for statistical analysis. Normal distribution continuous variables were expressed as the mean ± standard distribution.

## Results

### Patients baseline data

We collected data on 214 consecutive patients who underwent peripheral femoral cannulation. Mean age of patients was 51.03 ± 12.29 (range 23–72) years and 55.61% (119 of 214) were female. Mean BMI was 24.34 ± 1.99 kg/m^2^. Forty-eight patients (22.43%) were diagnosed with hypertension, twenty-eight (13.08%) patients with diabetes. The mean LVED was 60.31 ± 8.43 mm. The mean LVEF was 53.75 ± 7.33%. The baseline data are listed in Table 1. All procedures were electively performed.

**Table 1:** Baseline data

Item	Data
Male/Female	95/119
Age (years)	51.03 ± 12.29
BMI (kg/m^2^)	24.34 ± 1.99
Hypertension (n, %)	48 (22.43%)
Diabetes (n, %)	28 (13.08%)
LVED (mm)	60.31 ± 8.43
LVEF (%)	53.75 ± 7.33%

### Outcomes

All patients using this technique had successful peripheral cannulation. Femoral artery cannulation was carried out for totally endoscopic cardiac surgery in all patients. Of these, 201 were femoral vein cannulation and 13 were combined venous cannulations. Indications for surgery included mitral valve disease in 82.71% (177/214), atrial septal defect in 11.68% (25/214) and tricuspid regurgitation in the remaining 5.61% (12/214). Mean CPB time was 146.32 ± 31.28 mins. The detailed operative data are shown in Table 2. The in-hospital survival was 98.60% (Table 3). One died of renal failure following postoperative hemorrhage and secondary multiple organ failure, and two died of uncontrollable severe pneumonia and sepsis. There were no cases of intra-operative femoral arterial injuries and no dilator-related problems during surgery. There were no recorded cases of postoperative lower limb ischaemia or stroke. No cases of early or late wound infection. Wound seroma happened in only two patients. Only one (0.47%) patient showed clinical signs of venous thromboembolism. The femoral vein stenting was successfully performed and the patient was discharged from hospital. There was not a single case of late pseudoaneurysm present at the cannulation site.

**Table 2:** Intra-operative data

Item	Data
Surgery strategy	
Mitral valve surgery (n, %)	177 (82.71%)
Tricuspid valve surgery (n, %)	12 (5.61%)
Atrial septal defect repair (n, %)	25 (11.68%)
Cannulation site	
Femoral artery	214
Femoral venous	201
Combined venous	13
CPB time (min)	146.32 ± 31.28

**Table 3:** Postoperative Data

Item	TA group
Mortality	3 (1.40%)
Intensive care unit stay (hours)	32.13 ± 18.61
Stroke (n, %)	0
Lower limb ischaemia (n, %)	0
Embolism (n, %)	1 (0.47%)
Bleeding events (n, %)	2 (0.93%)
Groin wound problem (n, %)	2 (0.93%)
Pneumothorax (n, %)	4 (1.87%)
Subcutaneous emphysema (n, %)	3 (1.40%)

## Discussion

Since Mohr and Felger reported the initial successful experience of port-access cardiac surgery. Using single-lung ventilation, with CPB established through peripheral femoro-femoral cannulation and an endo-aortic balloon catheter for cardioplegia delivery and root de-airing. Different minimally invasive surgical approaches allowed heart valve surgery sparing sternal incision [3, 21]. Totally Endoscopic cardiac surgery is gradually popularized worldwide due to the cosmetic benefits of minimally invasive techniques with smaller incisions and faster recovery [3, 5, 22].

The controversy continues on the optimal cannulation site for totally endoscopic cardiac surgery. Best cannulation strategy for endoscopic surgery has been a topic of debate for nearly two decades. Cannulation technique to establish CPB during endoscopic surgery should be fast and using specific technique that minimizes the tissue trauma which could lead to the risk of reoperation, bleeding and need for blood transfusion. Malpositioned cannula may lead to low CPB flows, and inadequate drainage. Improper movement of the cannulas or poor secured cannula may lead to catastrophic decannulation. There are various approaches to establish cardiopulmonary bypass for endoscopic surgery. Because totally endoscopic cardiac surgery does not destroy the thoracic structure, extracorporeal circulation can be established through peripheral femoro-femoral cannulation.

Femoral artery cannulation has been routinely used since the early days of minimally invasive port-access mitral valve surgery [21, 23]. But negative neurocognitive outcomes and retrograde dissection associated with femoral retrograde perfusion has made peripheral approach decreasingly popular in this years [24–26], it finally being replaced by the ascending aorta canulation. This is despite the fact that cannulation of the distal ascending aorta is undoubtedly the most expedient and safest site for arterial inflow. However, with considerable advancements in minimally invasive techniques, including remarkable improvements in the quality of cannula and case selection under CT-guided, it seems that the use of retrograde femoral perfusion in totally endoscopic or robotic cardiac surgeries as safe and increasingly routine [27–29].

With the development of large-bore, thin-walled venous cannula with high flow and the improved performance of ultrasound machines have made femoral venous cannulation more popular. The single two-stage venous cannula is now selected as our primary choice. If the venous drainage is not smooth, the vacuum-assisted device can be initiated, and the internal jugular vein also can be cannulated during surgery. In addition, combined cannulation can be chosen in patients with interatrial sulcus opening, preoperative ultrasound indicated insufficient venous diameter or in-operative difficult exposure is expected. The literature shows that femoral vein cannulation is also very safe [30].

In our experience, peripheral femoro-femoral cannulation for retrograde perfusion and venous drainage from the inferior vena cava remains a good option. It needs to follow the below fundamental principles: (1) rapid establishment of CPB and maintenance of adequate perfusion, (2) optimal exposure and make sure the exposure is not affected, and (3) suitable for the patient’s pathology. Cannulation under direct vision is simple in anatomy and reduces the risks associated with percutaneous cannulation. These include difficulty in placing large cannulaes, rupture of vessels, and retroperitoneal hematoma. The procedure is simple and fast, and there is no need for complex manipulating of the femoral artery and vein (such as side-arm), and there are few local complications.

Here, we present a description of the experience of 214 consecutive patients who received open Seldinger-guided peripheral femoro-femoral cannulation for totally endoscopic cardiac surgery. This technique is rapid and simple, only minimal manipulation and dissection of the femoral vessels is required. Our results strongly support the use of this technology. The overall survival rate of the patients was 98.60%. None of the deaths were associated with femoral vascular cannulation and retrograde perfusion of the femoral artery. This rate is similar with a series of recently published reports [25, 31–33]. In addition, we had no patients with stroke and we found no cases of inadequate CPB flow, aortic dissection or arterial injury. The additional benefit of not requiring an arterial snare, which allows distal limb perfusion, is also supported by our results. No one of lower limb ischemia was identified. Wound seroma happened in only two patients, and our wound complication rate was 0.93%.

The excellent outcome of our peripheral femoro-femoral cannulation using the open Seldinger-guided technique has several potential reasons. Firstly, an important adjunct to this is the pre-operative CT scan and the intra-operative use of ultrasound, which allows us to identify patients with iliac and femoral atheromatous plaques, which could potentially mobilize aortic debris to enter the cerebral circulation, which may increase retrograde perfusion cerebrovascular complications. Secondly, the simple manipulation and minimal dissection of the femoral vessels minimize local vascular trauma and reduce perivascular lymphatic destruction, so wound infection and seroma are minimized. Thirdly, the large-bore, thin-walled cannula incorporating a tapered tip, which has a smooth dilator-to-cannula transition, not only minimizes the size of the vascular opening but also allows easy insertion with the Seldinger-guided technique, which could be repaired by simply tying the purse-string.

Other forms of femoral artery cannulation that have become quite popular are not without their obvious disadvantages. As it is more time consuming, the use of side-arm grafts is contraindicated. In addition, the side-arm grafts anastomosis is prone to bleed during the CPB. The side-arm must be suture to close at the end of the CPB. The permanent graft will remain in place indefinitely. Peripheral extracorporeal circulation through the femoral vessels is an accepted technique. In contrast, open Seldinger-guided femoral cannulation simply and completely avoids these drawbacks associated with sidearm or incision technique. In the subject of venous cannulation, the single two stage venous cannula can avoid the jugular cannulation. With percutaneous puncture, venous drainage is impeded due to the small size of the cannula. Open Seldinger-guided femoro-femoral technique can avoid these drawbacks.

In discussing the limitations of this research, it is important to indicate that this study is not an attempt to compare different peripheral cannulation techniques. It is a retrospective study that our goals are simply to indicate the efficacy and safety of this technique for peripheral femoral cannulation.

## Conclusion

This study indicates that the open Seldinger-guided femoro-femoral technique for peripheral femoral cannulation is efficient and safe under direct vision and with intra-operative TEE. The surgical trauma of the femoral vessel is minimized. Using the Seldinger-guided technique decreases the manipulate time. Besides, the vessel is not snared, thereby preserving the distal blood flow and reducing the risk of limb ischaemia. We recommend open Seldinger-guided peripheral femoral cannulation for its rapidity, simplicity and effectiveness.

## Supplementary Information

**Additional file 1.**

**Additional file 2.**

**Additional file 3.**

**Additional file 4.**

## Data Availability

Data sharing was not applicable to this article, as no data sets were generated or analysed during the current study.

## Abbreviations

MICS: Minimally invasive cardiac surgery
CPB: Cardiopulmonary bypass
TEE: Transesophageal echocardiography
