# Supplementary figures and images for: Open Seldinger-guided peripheral femoro-femoral cannulation technique for totally endoscopic cardiac surgery
Source: J Cardiothorac Surg. 2021 Jul 22;16:199. doi: 10.1186/s13019-021-01584-x (PMC8296695; doi:10.1186/s13019-021-01584-x)

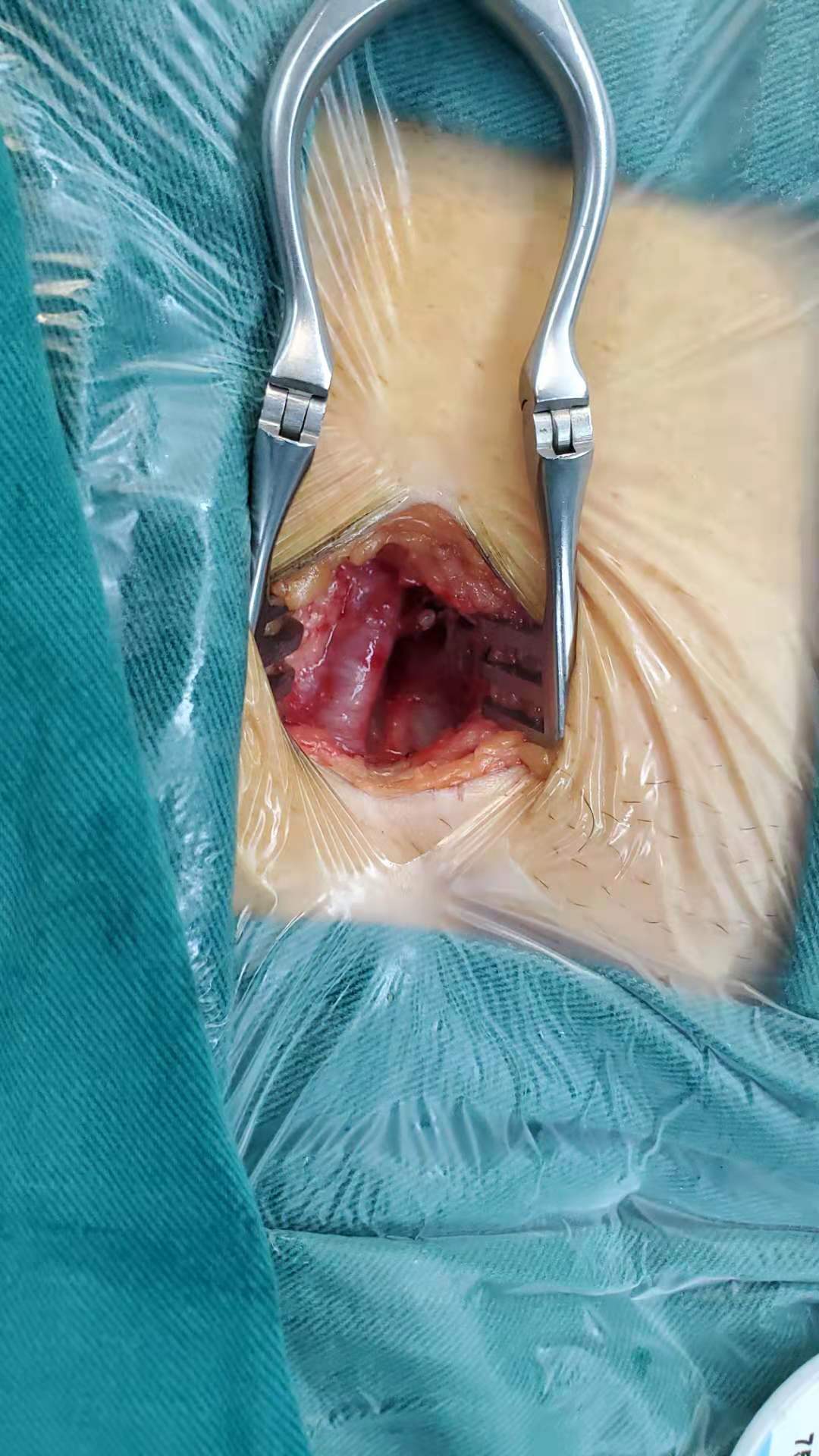

Supplement: Supplementary file 1 — Additional file 1. [file 13019_2021_1584_MOESM1_ESM.jpg]

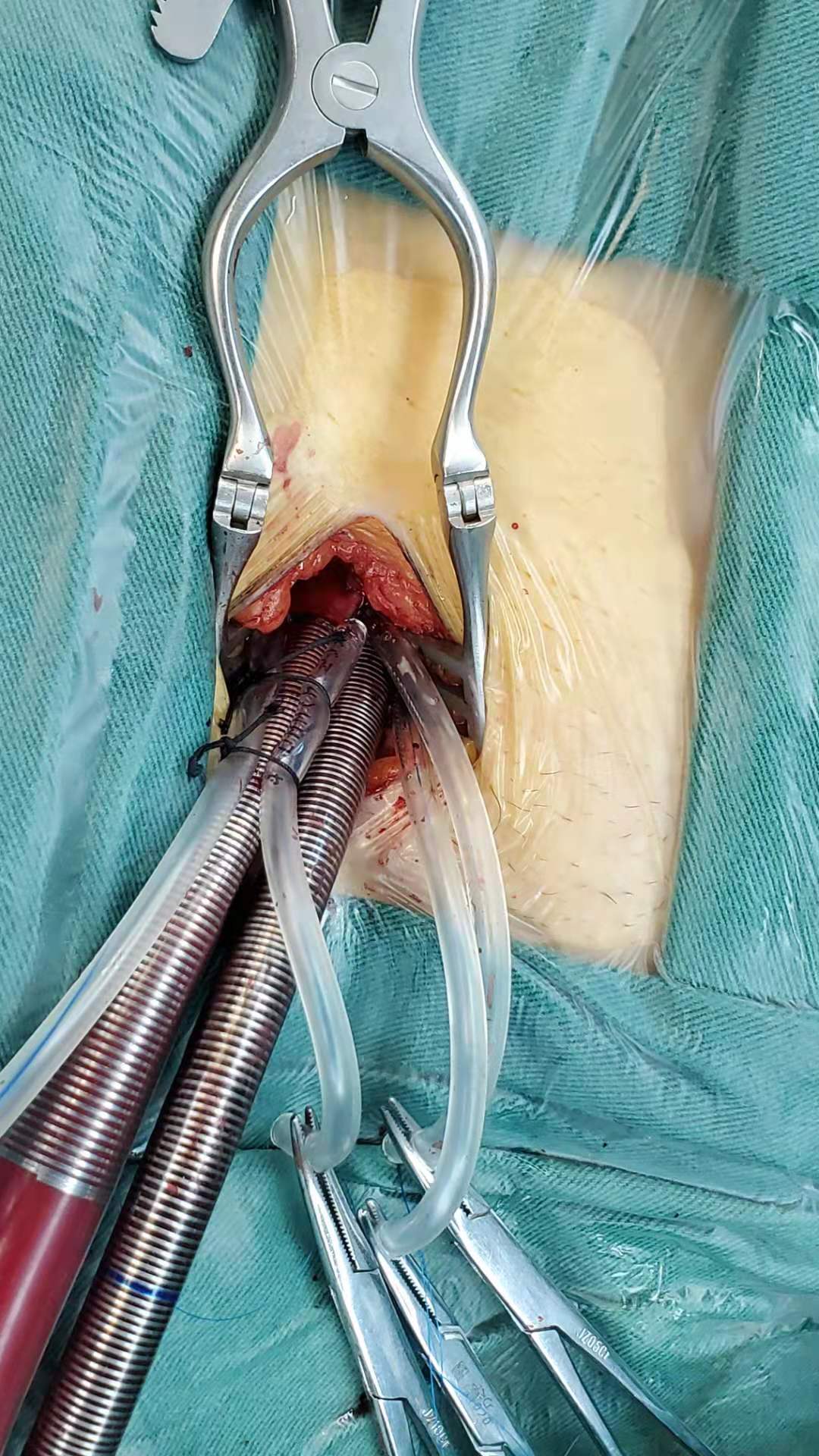

Supplement: Supplementary file 2 — Additional file 2. [file 13019_2021_1584_MOESM2_ESM.jpg]

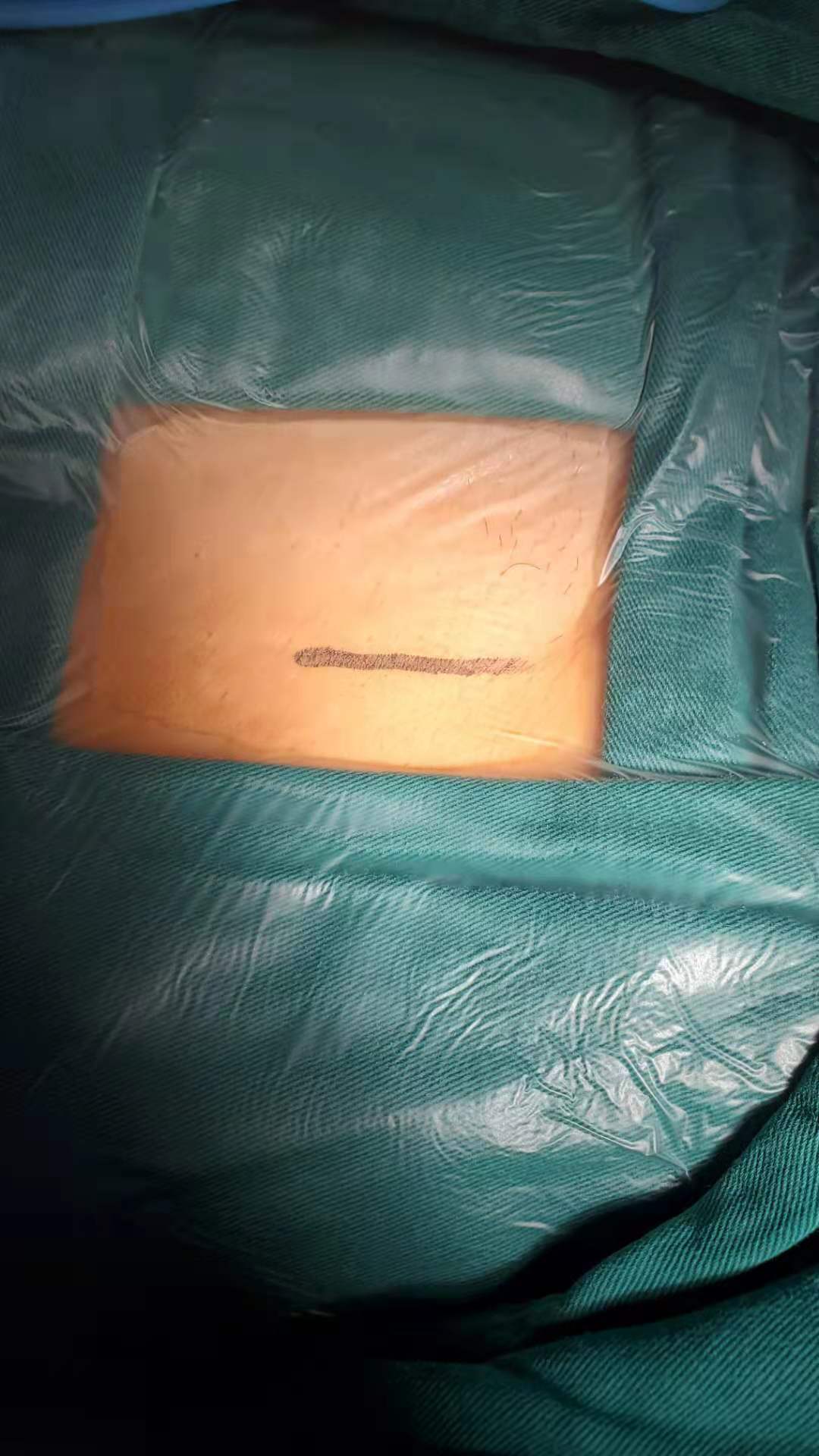

Supplement: Supplementary file 3 — Additional file 3. [file 13019_2021_1584_MOESM3_ESM.jpg]

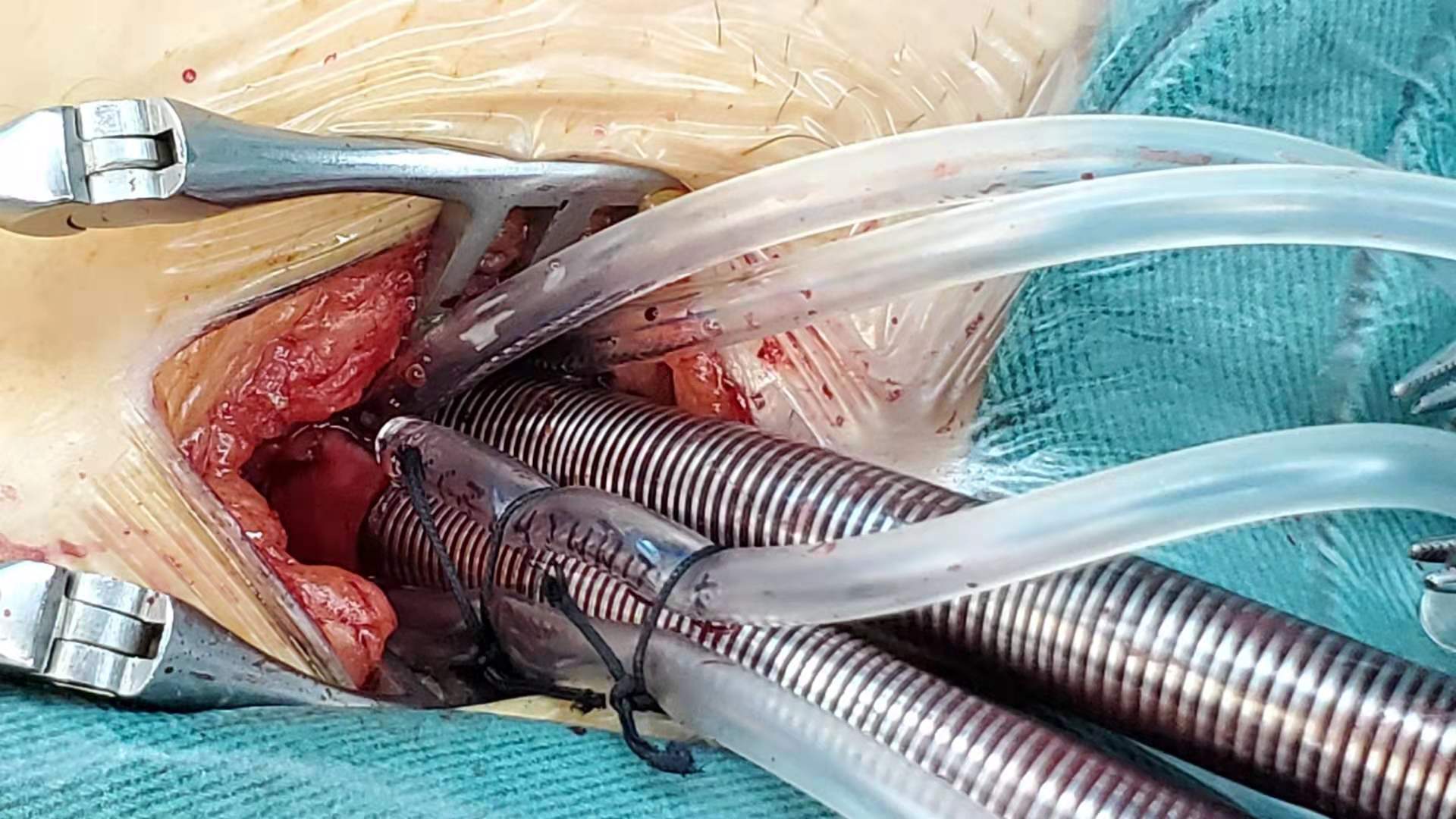

Supplement: Supplementary file 4 — Additional file 4. [file 13019_2021_1584_MOESM4_ESM.jpg]
